# Ratiometric Bioluminescent Zinc Sensor Proteins to Quantify Serum and Intracellular Free Zn^2+^

**DOI:** 10.1021/acschembio.2c00227

**Published:** 2022-05-25

**Authors:** Claire M. S. Michielsen, Eva A. van Aalen, Maarten Merkx

**Affiliations:** ^†^Laboratory of Chemical Biology, Department of Biomedical Engineering and ^‡^Institute for Complex Molecular Systems, Eindhoven University of Technology, P.O Box 513, 5600 MB Eindhoven, The Netherlands

## Abstract

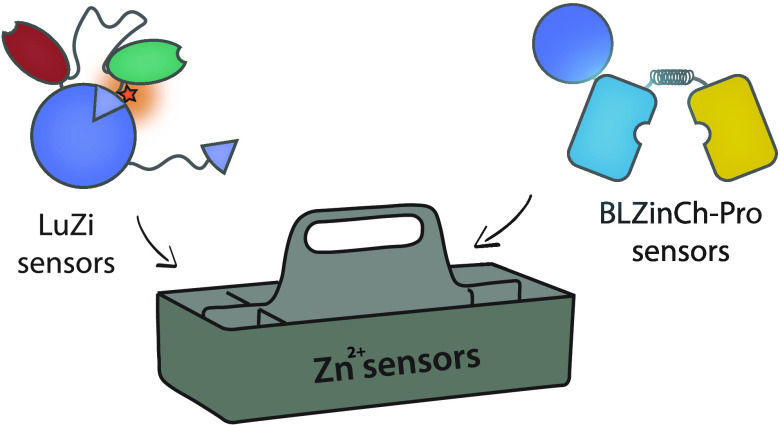

Fluorescent Zn^2+^ sensors play a pivotal role in zinc biology, but their application in complex media such as blood serum or plate reader-based cellular assays is hampered by autofluorescence and light scattering. Bioluminescent sensor proteins provide an attractive alternative to fluorescent sensors for these applications, but the only bioluminescent sensor protein developed so far, BLZinCh, has a limited sensor response and a suboptimal Zn^2+^ affinity. In this work, we expanded the toolbox of bioluminescent Zn^2+^ sensors by developing two new sensor families that show a large change in the emission ratio and cover a range of physiologically relevant Zn^2+^ affinities. The LuZi platform relies on competitive complementation of split NanoLuc luciferase and displays a robust, 2-fold change in red-to-blue emission, allowing quantification of free Zn^2+^ between 2 pM and 1 nM. The second platform was developed by replacing the long flexible GGS linker in the original BLZinCh sensor by rigid polyproline linkers, yielding a series of BLZinCh-Pro sensors with a 3–4-fold improved ratiometric response and physiologically relevant Zn^2+^ affinities between 0.5 and 1 nM. Both the LuZi and BLZinCh-Pro sensors allowed the direct determination of low nM concentrations of free Zn^2+^ in serum, providing an attractive alternative to more laborious and/or indirect approaches to measure serum zinc levels. Furthermore, the genetic encoding of the BLZinCh-Pro sensors allowed their use as intracellular sensors, where the sensor occupancy of 40–50% makes them ideally suited to monitor both increases and decreases in intracellular free Zn^2+^ concentration in simple, plate reader-based measurements, without the need for fluorescence microscopy.

## Introduction

Zn^2+^ is an essential trace element that plays a key role in many biochemical processes, including enzyme catalysis, the regulation of gene expression, and intracellular signaling.^1−4^ Given this importance, it is not surprising that zinc deficiency is associated with severe risk of, among others, growth retardation, neural dysfunctions, and imbalanced immune responses.^5−9^ Approximately 17% of the global population is at risk of acquiring zinc deficiency, in particular in low- and middle-income countries,^10,11^ resulting in millions of disability-adjusted life years and thousands of deaths among children under the age of five, every year.^12−14^ Zinc deficiency is currently determined by measuring the total serum Zn^2+^ concentration (12–16 μM in healthy people) using expensive and technically complex instruments such as atomic absorption spectroscopy (AAS) and inductively coupled plasma mass spectrometry (ICP-MS).^15,16^ In addition, it is unclear whether total serum Zn^2+^ represents a good measure of the zinc status, as almost all serum Zn^2+^ is tightly bound to plasma proteins, such as albumin (80–90%) and α-2-macroglobulin (10–20%).^17−19^ The concentration of free Zn^2+^ may be a more physiologically relevant biomarker, since it represents the bioavailable part of the serum Zn^2+^.^19^ However, simple and robust methods to measure the concentration of free Zn^2+^ in serum and complex biological matrices are currently lacking.

**Figure 1:**
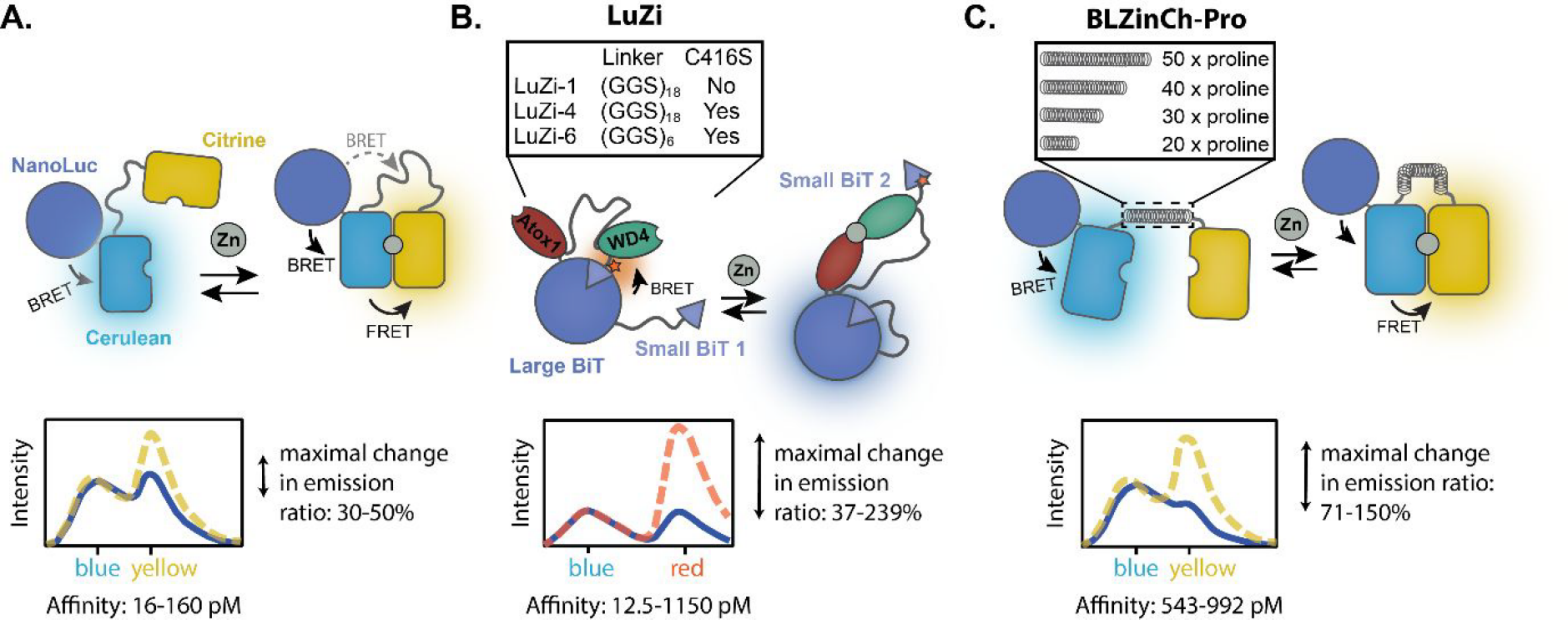
Bioluminescent Zn^2+^ sensor protein platforms. (A) Schematic representation of the concept of free Zn^2+^ detection using the previously developed BLZinCh sensor proteins. BRET occurs from NanoLuc (NLuc) to Cerulean, and in the presence of free Zn^2+^, the Zn^2+^-coordinating residues on the exterior of Cerulean and Citrine (connected via a flexible GGS linker) bind to Zn^2+^, and FRET occurs from Cerulean to Citrine. This results in the emission of yellow light. The BLZinCh sensors have a moderate maximal change in the emission ratio (30–50%) and a suboptimal, high affinity for Zn^2+^ (16–160 pM). (B) Overview of the LuZi platform. The LuZi sensor proteins contain the two Zn^2+^-binding domains Atox1 and WD4 and have a readout mechanism based on split NLuc complementation. The NLuc large BiT (LB) is fused to two small BiTs (SB), with different affinities for LB. A red-emitting Cy3 fluorophore (red star) is conjugated to the higher affinity SB (SB2), resulting in a high BRET efficiency in the absence of Zn^2+^. Binding of Zn^2+^ disrupts the interaction of SB2 to LB, changing the emission from red to blue. (C) Overview of the BLZinCh-Pro platform, where the original flexible linker between Cerulean and Citrine in the BLZinCh sensors is replaced with four different rigid polyproline linkers (50x, 40x, 30x, and 20x proline). These rigid linkers increase the distance between the fluorescent proteins in the Zn^2+^-depleted state, resulting in an increased maximal change in the emission ratio and an attenuated affinity for Zn^2+^.

The development of small-molecule fluorescent probes and fluorescent sensor proteins that measure intracellular free Zn^2+^ concentrations has substantially contributed to our understanding of intracellular Zn^2+^ homeostasis and signaling.^20−25^ Genetically encoded, protein-based sensors have proven particularly useful as they can be applied to measure fluctuations in free Zn^2+^ concentrations at specific subcellular locations in live cells with minimal perturbation of the cell integrity. Most previously developed Zn^2+^ sensor proteins are based on the modulation of Förster Resonance Energy Transfer (FRET), resulting in a ratiometric sensor output that is critical for reliable free Zn^2+^ quantification. A rich toolbox of fluorescent sensor proteins has been developed, covering a wide range of Zn^2+^ affinities, colors, and subcellular targeting. For example, the eCALWY, Zap, and eZinCh2 families of Zn^2+^ sensors have been widely used to measure intracellular free Zn^2+^ concentrations in cell lines, primary cells, plants, and whole organisms.^21−23^ A drawback of fluorescence-based sensors is their dependence on external illumination, which hinders long-term imaging due to photobleaching and phototoxicity. Furthermore, fluorescent measurements in complex media such as blood serum or plate reader-based cellular assays are severely hampered by autofluorescence and light scattering. For these applications, bioluminescent sensor proteins, based on the modulation of energy transfer between a donor luciferase and an acceptor fluorescent domain (BRET), provide attractive alternatives. We previously developed a bioluminescent variant of the eZinCh2 sensor by introducing the luciferase NanoLuc (NLuc) at the N-terminus of the Cerulean fluorescent donor domain.^23,26,27^ The resulting BLZinCh-1 sensor allowed both fluorescent and bioluminescent detection, showing a modest 30% change in the bioluminescence emission ratio upon binding to Zn^2+^ (Figure 1A). The limited ratiometric response of the BLZinCh-1 sensor could be increased to 50% by introduction of a chromophore-silencing mutation in Cerulean, providing better spectral separation between the donor and acceptor emission. However, the resulting BLZinCh-3 sensor also showed an increase in Zn^2+^ affinity to *K*_D_ = 16 pM, which is suboptimal for measuring intracellular cytosolic Zn^2+^ concentrations that are typically in the 0.5–1 nM range.^22,23,28^

In this work, we expand the toolbox of bioluminescent Zn^2+^ sensor proteins by developing two new sensor formats that show a large change in the emission ratio and cover a range of physiologically relevant Zn^2+^ affinities. The first platform (LuZi, Figure 1B) uses the Zn^2+^-binding receptor domains previously employed in the eCALWY FRET sensors to control the competitive intramolecular complementation of a split NLuc luciferase between a high BRET, Zn^2+^-free state and a low BRET, Zn^2+^-bound state. A similar sensor principle was recently successfully introduced to increase the performance of BRET sensors for antibody detection, because it uses a red fluorescent acceptor that is well separated from the NLuc emission.^29^ The second platform (BLZinCh-Pro, Figure 1C) substantially improves upon the performance of the BLZinCh sensors by replacing the flexible linker separating the fluorescent domains by rigid polyproline linkers, simultaneously decreasing BRET in the Zn^2+^-free state and subtly attenuating the affinity into the more physiologically relevant ∼0.5–1 nM affinity range. These new sensors allow fast, robust, and sensitive quantification of free Zn^2+^ concentrations, both in blood serum samples and in the cytosol of mammalian cells.

## Results and Discussion

### LuZi: Red-Blue Bioluminescent Zn^2+^ Sensors Based on Split NanoLuc Complementation

In previous work, we already explored the development of bioluminescent variants of the eCALWY series of FRET sensors by fusing NLuc to the N-terminus of the Cerulean fluorescent domain.^27^ However, only very small changes in the BRET ratio were observed for these sensors (<7%), which is unfortunate as the Zn^2+^-binding part of the eCALWY sensors can be readily tuned to bind Zn^2+^ with a range of affinities between 2 pM and 5 nM.^22,30^ The poor performance of the eCALWY sensors is probably due to suboptimal energy transfer from NLuc to Cerulean and direct BRET between NLuc and Citrine in the absence of Zn^2+^. We therefore decided to take advantage of a new design principle that does not rely on direct modulation of BRET efficiency but is based on competitive intramolecular complementation of split NLuc.^29^ Split NLuc is composed of the 18 kDa large BiT (LB) fragment and the 1.3 kDa small BiT (SB) peptide whose interaction can be readily tuned between 0.7 nM and 190 μM.^31^ In the herein developed LuZi sensor, we genetically fused a single LB to two SB fragments, SB1 and SB2, with a low (*K*_D_ = 190 μM) and moderate (*K*_D_ = 2.5 μM) affinity for LB, respectively. In the Zn^2+^-depleted state, LB can form a complex with the high affinity SB2, which allows efficient BRET from the complemented NLuc to the red-emitting Cy3 dye conjugated next to SB2. Upon Zn^2+^ binding to the Atox1 and WD4 Zn^2+^-binding domains, the interaction between SB2 and LB is disrupted, allowing for subsequent formation of a complex between LB and SB1, which produces predominantly blue light.

**Figure 2:**
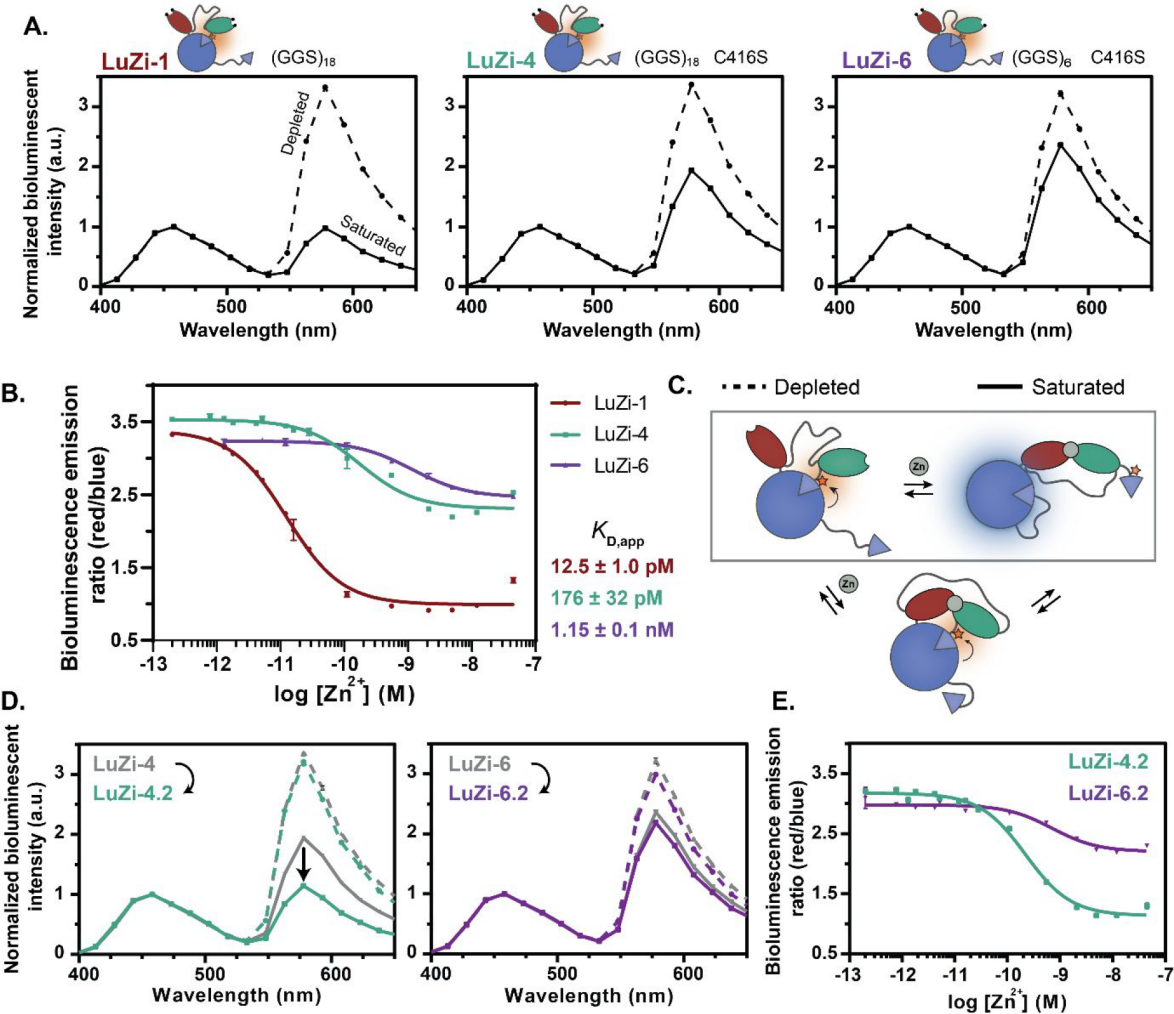
Performance and optimization of the LuZi sensor proteins. (A) Bioluminescence emission spectra (normalized to emission at 458 nm) of LuZi-1, LuZi-4, and LuZi-6 in the Zn^2+^-depleted (dashed line) and Zn^2+^-saturated (solid line) state. (B) Bioluminescence emission ratio (578 nm/458 nm) of the LuZi variants in the presence of a range of free Zn^2+^ concentrations buffered using 1 mM HEDTA, 1 mM DHPTA, or 1 mM EGTA. LuZi-1, LuZi-4, and LuZi-6 yielded *K*_D,app_ values of 12.5 ± 1.0 pM, 176 ± 32 pM, and 1.15 ± 0.1 nM, respectively (Supplementary Table 2). (C) Schematic representation of our hypothesis of the two possible sensor conformations in the presence of Zn^2+^, explaining the high BRET in the Zn^2+^-saturated state. (D) Bioluminescence emission spectra (normalized to emission at 458 nm) of LuZi-4.2 and LuZi-6.2 in the Zn^2+^-depleted (dashed line) and Zn^2+^-saturated (solid line) state. The gray spectra represent the original sensors (LuZi-4 and LuZi-6), and the colored spectra represent the optimized variants (LuZi-4.2 and LuZi-6.2). (E) Bioluminescence emission ratio (578 nm/458 nm) of LuZi-4.2 and LuZi-6.2 as a function of free Zn^2+^ concentration. LuZi-4.2 and LuZi-6.2 yielded *K*_D,app_ values of 221 ± 18 pM and 708 ± 147 pM, respectively. Measurements were performed using 2 nM sensor protein and 1000-fold diluted NLuc substrate in 150 mM HEPES (pH 7.1), 100 mM NaCl, 10% (v/v) glycerol, 5 μM DTT, 1 mM TCEP, and 1 mg mL^–1^ BSA at 20 °C. Error bars represent average ± s.d. (*n* = 3), and the solid lines are fitted using eq 3 (Methods section).

To cover a range of different Zn^2+^ affinities, we designed three LuZi variants based on the Zn^2+^-binding domains of eCALWY-1 (*K*_D_ = 2 pM), eCALWY-4 (*K*_D_ = 630 pM), and eCALWY-6 (*K*_D_ = 2900 pM) to yield LuZi-1, LuZi-4, and LuZi-6, respectively. The presence of cysteines in the Zn^2+^-binding domains precluded the use of cysteine-maleimide chemistry to introduce the Cy3 dye. Therefore, the noncanonical amino acid *p*-azidophenylalanine (pAzF) was introduced next to the SB2 domain to allow site-specific introduction of the Cy3 dye via strain-promoted azide–alkyne click chemistry (SPAAC). Expression of the different LuZi variants was successfully performed in *Escherichia coli* with amber codon suppression using an orthogonal tRNA synthetase/tRNA pair for the incorporation of the pAzF. After affinity chromatography purification, this noncanonical amino acid was used to conjugate a DBCO-functionalized Cy3 dye, yielding a ∼75% degree of labeling (Figure S1). LuZi-1 displayed a high red emission in the absence of Zn^2+^, ensuing from efficient BRET from reconstituted NLuc to the Cy3 dye (Figure 2A). Addition of Zn^2+^ resulted in a large decrease in the red/blue emission ratio, which is consistent with Zn^2+^-binding induced disruption of the LB-SB2 interaction and subsequent formation of the LB-SB1 complex. Zn^2+^ titration experiments with LuZi-1 showed a 239% maximal change in the emission ratio upon Zn^2+^ binding and yielded a *K*_D_ of 12.5 ± 1.0 pM, which is similar to that of the eCALWY-1 sensor (Figure 2B). LuZi-4 and LuZi-6 also showed a Zn^2+^ induced decrease in the red/blue emission ratio, although the change in the emission ratio was attenuated compared to that of LuZi-1 (74% and 36%, respectively). Titration experiments yielded Zn^2+^-binding affinities of 176 ± 32 pM and 1.15 ± 0.1 nM for LuZi-4 and LuZi-6, respectively, which again are comparable to the Zn^2+^ affinities of their parental eCALWY sensors (Figure 2B). The relatively modest changes in the emission ratio observed for LuZi-4 and LuZi-6 are primarily due to the high Cy3 emission (578 nm) in the Zn^2+^-saturated state, which suggests that SB2 might still partially interact with LB in this state (Figure 2C). The only difference between LuZi-1 and LuZi-4 is the replacement of one of the four Zn^2+^-coordinating cysteines in LuZi-1 by a serine in LuZi-4. This substitution not only weakens the Zn^2+^ affinity, but coordination by three cysteines apparently also allows for a conformation that is still compatible with an LB-SB2 interaction, whereas coordination by four cysteines in LuZi-1 results in a conformation that is incompatible with the LB-SB2 interaction. We hypothesized that by reducing the freedom of movement of the Zn^2+^-binding domains (Atox1 and WD4) in the Zn^2+^-saturated state of LuZi-4 and LuZi-6, SB2 could be prevented from forming a complex with LB, thus suppressing undesired BRET in the saturated state of the sensor. To do so, we deleted four amino acids in the linker between Atox1 and five amino acids in the linker between WD4 and SB2 (Figure S8). The resulting LuZi-4.2 and LuZi-6.2 variants were successfully expressed, purified, and conjugated with Cy3, showing labeling efficiencies of 84% and 63%, respectively (Figure S1). For LuZi-4.2, a substantial decrease in BRET was indeed observed for the Zn^2+^-saturated state compared to that of LuZi-4, resulting in an increase in the ratiometric response from 74% to 181% obtaining a similar *K*_D*,app*_ of 221 ± 18 pM (Figures 2D and 2E). Unfortunately, shortening of the linkers did not improve the ratiometric response of the LuZi-6 sensor, with the LuZi-6.2 sensor showing emission spectra that are very similar to that of LuZi-6 (Figures 2D and 2E). Furthermore, the affinity of LuZi-6.2 for Zn^2+^ had slightly decreased (*K*_D*,app*_ = 708 ± 147 pM). Nonetheless, two attractive bioluminescent sensor proteins were obtained (LuZi-1 and LuZi-4.2) that show a robust, 2-fold change in the emission ratio and together allow quantification of free Zn^2+^ concentrations between 2 pM and 1 nM.

### BLZinCh-Pro: Improving Sensor Performance by Introduction of Polyproline Linkers

The previously developed BLZinCh-1 sensor consisted of two fluorescent protein domains containing exterior Zn^2+^-coordinating residues, connected via a long linker with 18 GGS repeats. The flexibility of this linker allowed the fluorescent proteins to form a complex in the presence of Zn^2+^, resulting in efficient FRET in the Zn^2+^-bound state. In the Zn^2+^-depleted state, this sensor adopts an ensemble of different conformations. Although the average distance between the fluorescent domains is larger in the Zn^2+^-depleted state than in the Zn^2+^-bound state, flexible linkers form relatively compact ensembles, giving rise to a substantial amount of energy transfer also in the Zn^2+^-depleted state.^32^ Due to the high energy transfer efficiency in the Zn^2+^-bound state, this was not a problem when measuring FRET (ratiometric change of ∼400%), but it severely affected the BRET response.^23^ Therefore, to increase the distance between the fluorescent proteins in the Zn^2+^-depleted state, we developed the BLZinCh-Pro series of bioluminescent Zn^2+^ sensors in which the flexible (GGS)_18_ linker was replaced by rigid polyproline linkers of different lengths. We first incorporated a 50x proline linker (BLZinCh-P50) in the BLZinCh-1 backbone and subsequently used digestion with the restriction enzyme BseRI to generate three other variants: BLZinCh-P20, BLZinCh-P30, and BLZinCh-P40, with respectively 20, 30, or 40 prolines in the linker (Figures 1C, S3, S6, and S7).

**Figure 3:**
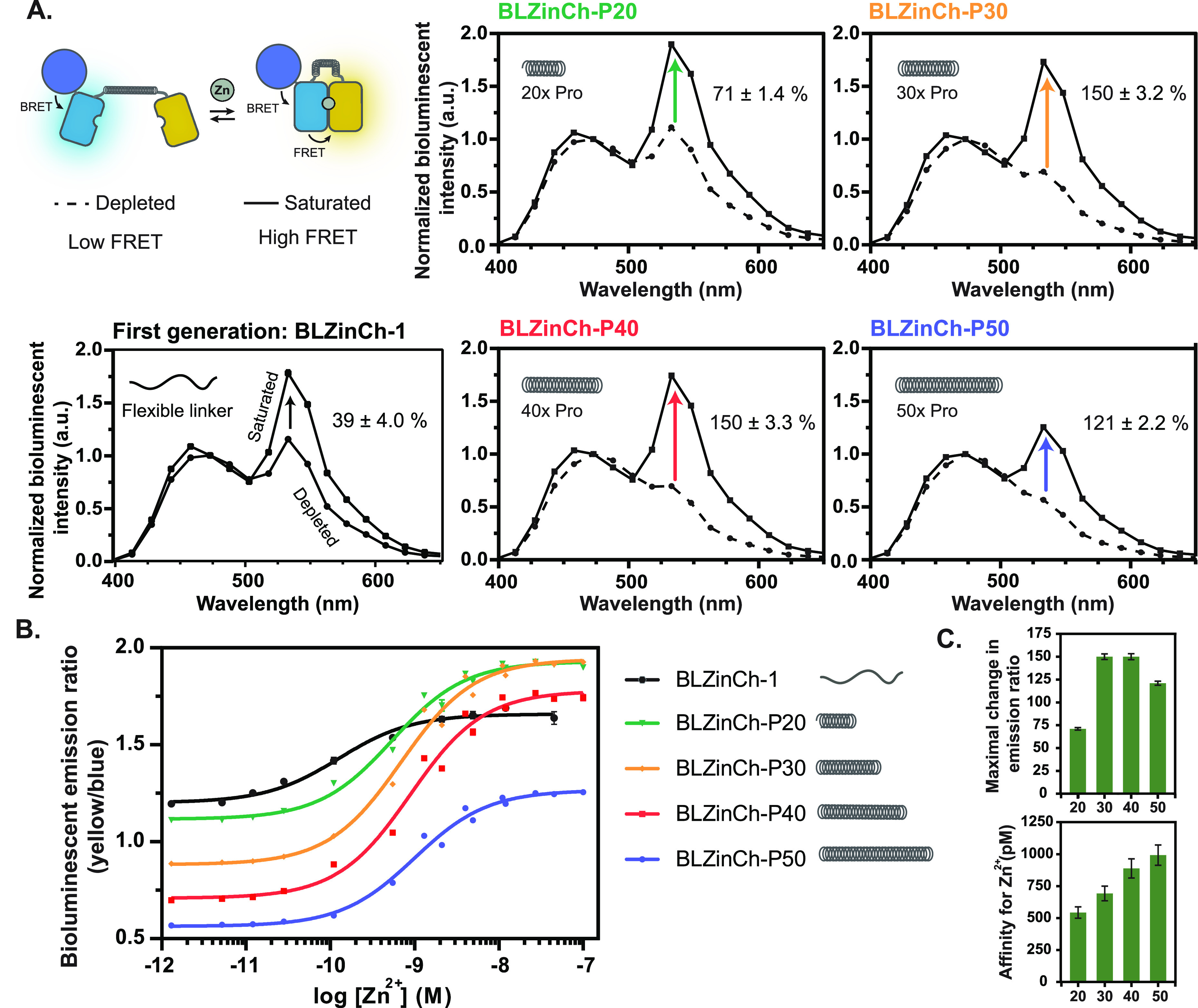
Performance of the BLZinCh-Pro sensor proteins. (A) Bioluminescence emission spectra (normalized to emission at 475 nm) of the BLZinCh-1 and BLZinCh-Pro variants in the Zn^2+^-depleted (dashed line) and Zn^2+^-saturated (solid line) state. (B) Bioluminescence emission ratio (533 nm/473 nm) of the BLZinCh-Pro variants in the presence of a range of free Zn^2+^ concentrations buffered using 1 mM DHPTA, 1 mM EGTA, or 1 mM NTA. Measurements were performed using 0.2 nM sensor protein and 2000-fold diluted NLuc substrate in 150 mM HEPES (pH 7.1), 100 mM NaCl, 10% (v/v) glycerol, 5 μM DTT, 1 mM TCEP, and 1 mg mL^–1^ BSA at 20 °C (Supplementary Table 2). Each data point is the average of three measurements ± standard deviation (s.d.), and the solid lines are fitted using eq 3 (Methods section). (C) Maximal change in the emission ratio and affinity for Zn^2+^ of each BLZinCh-Pro variant. Bars represent average values ± standard deviation (s.d.).

Following successful expression and purification of the BLZinCh-Pro sensors (Figure S2), bioluminescent spectra were obtained for all variants in the absence and presence of Zn^2+^. In the Zn^2+^-free state, a consistent decrease of the Citrine emission (533 nm) was observed upon increasing the linker length from 20 to 50 proline residues, with no or very little Citrine emission observed for the sensors with the longer linkers (Figure 3A). This shows that the two fluorescent domains are more effectively separated in the BLZinCh-Pro sensors compared to the parent BLZinCh-1 sensor, reducing undesired FRET and BRET in the absence of Zn^2+^. In contrast, very similar bioluminescence spectra were obtained in the presence of saturating amounts of Zn^2+^ for BLZinCh-1, BLZinCh-P20, BLZinCh-P30, and BLZinCh-P40, while a somewhat lower ratio was observed for BLZinCh-P50. These results show that the BLZinCh-1 and BLZinCh-Pro sensors form the same Zn^2+^-bound complex despite the more rigid linkers in the latter, with the exception of BLZinCh-P50 where mechanical strain may affect the relative orientation of the fluorescent domains or preclude complete formation of the closed, high-FRET/BRET state. As a result, introduction of the different proline linkers resulted in an increased ratiometric response from 39% in BLZinCh-1 to 71%, 150%, 150%, and 121%, for BLZinCh-P20, -P30, -P40, and -P50, respectively (Figure 3A). Introduction of the proline linkers also attenuated the Zn^2+^ affinity of the sensors, yielding *K*_D,app_ of 543 ± 45 pM, 693 ± 57 pM, 889 ± 76 pM, and 992 ± 80 pM for BLZinCh-P20, BLZinCh-P30, BLZinCh-P40, and BLZinCh-50, respectively (Figures 3B and 3C). The attenuation of the Zn^2+^ affinity can be understood by the effect of linker stiffness and linker length on the effective concentration for complex formation. Indeed, for the 20–30 Å distance between the linker ends in the Zn^2+^-bound state of the sensor, higher effective concentrations are predicted for flexible linkers compared to polyproline linkers.^32,33^ Furthermore, a decrease in effective linker length is expected upon increasing the length of the stiff polyproline linker, which is in line with the small but consistent increase in *K*_D_ observed experimentally when increasing the length of the proline linker. Importantly, the attenuation of the Zn^2+^ affinity from 160 pM in the parental BLZinCh-1 to the 0.5–1 nM affinities for the BLZinCh-Pro sensors makes the latter better suited for measuring intracellular cytosolic Zn^2+^ and free Zn^2+^ in serum.

### Bioluminescent Measurement of Free Zn^2+^ Concentration in Serum

The robust change in the emission ratio of the LuZi and BLZinCh-Pro sensors and their Zn^2+^ affinities in the 0.1–1 nM range make these bioluminescent sensors attractive tools for measuring free Zn^2+^ concentrations in blood plasma and serum. To date, the determination of free Zn^2+^ concentrations in plasma and serum has proven to be challenging, with previous approaches relying on either indirect measurements based on the activity of Zn^2+^-dependent reporter enzymes or the use of fluorescent dyes.^34^ For example, Magneson and co-workers used the activity of the enzyme phosphoglucomutase to derive a free Zn^2+^ concentration of 0.2 nM in blood plasma.^35^ Using the ZnAF-2 fluorescent sensor dye, Soybel and co-workers reported free Zn^2+^ concentration in rat serum between 1 and 3 nM,^36^ whereas studies with Zinpyr-1 by Haase and co-workers yielded free Zn^2+^ concentration of 0.22 ± 0.05 nM in human serum.^34^ Fluorescent dyes such as ZinPyr-1 and ZnAF-2 may suffer from interaction with other blood components such as serum albumin, which could affect their sensor properties.^37−39^ To apply the herein developed bioluminescence-based sensors for the quantification of free Zn^2+^ in human blood serum, we first assessed the performance of the LuZi sensors. To compare our results with the recent study of Haase and co-workers using ZinPyr-1, we performed all measurements in 2% (v/v) serum in buffer. Accordingly, we measured the emission ratio of LuZi-1, LuZi-4.2, and LuZi-6.2 in 2% human serum in the absence and presence of 50 mM EDTA to scavenge all Zn^2+^ or in the presence of a saturating amount of Zn^2+^. For the latter, addition of 8 μM ZnCl_2_ was found to be optimal, as addition of higher concentrations resulted in an emission ratio similar to that of the Zn^2+^ depleted state (Figure S5). Our explanation for this surprising finding is that at high Zn^2+^ concentrations binding of Zn^2+^ to each of the metal-binding domains, Atox1 and WD4, becomes favorable over coordination of a single Zn^2+^ between the two domains. The free Zn^2+^ concentrations can then be calculated from the measured emission ratios using
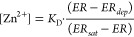
1where *ER*, *ER*_*dep*_, and *ER*_*sat*_ represent the emission ratios in the unaltered, Zn^2+^-depleted, and Zn^2+^-saturated state, respectively.

**Figure 4:**
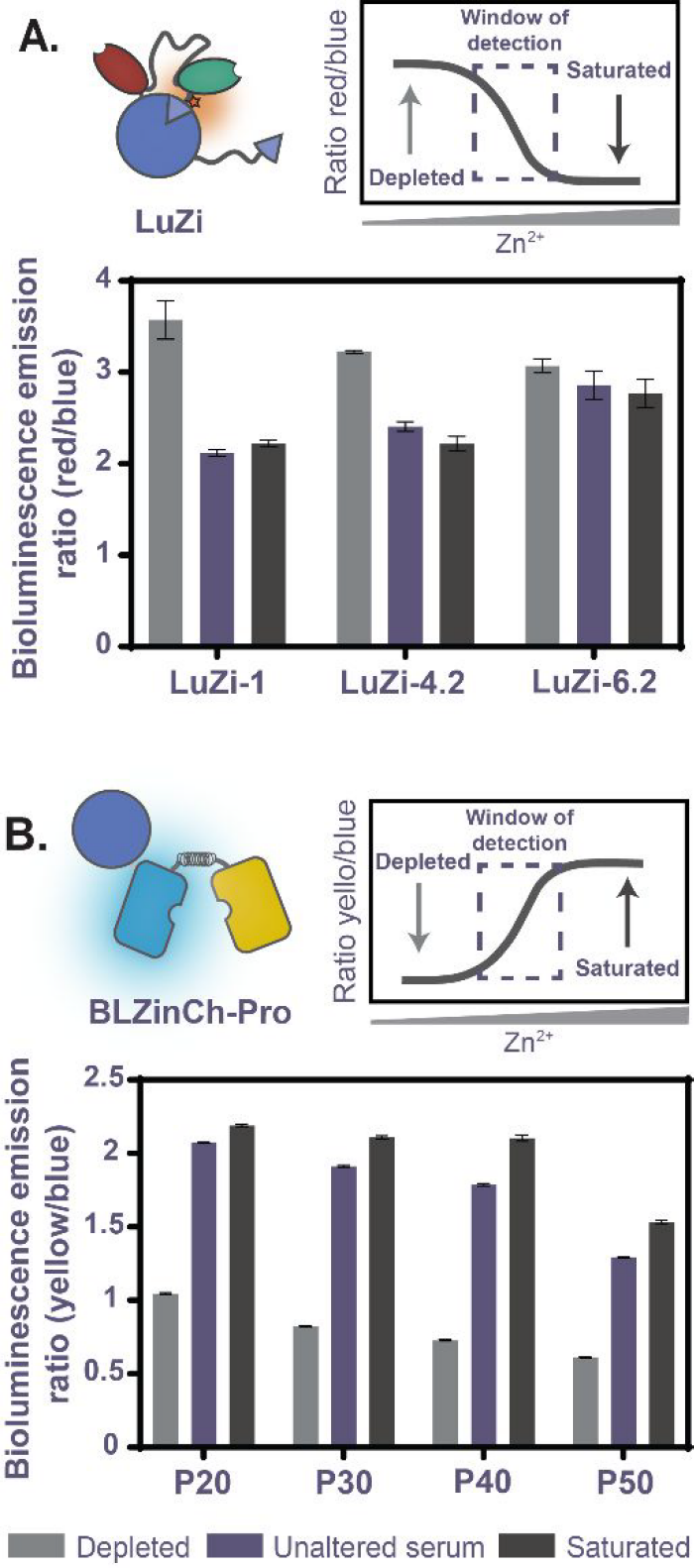
Determination of free Zn^2+^ concentration in diluted human serum. Bioluminescence emission ratio of (A) the LuZi sensors (578 nm/458 nm) and (B) BLZinCh-Pro sensors (533 nm/ 473 nm) in 2% (v/v) human serum in buffer. The Zn^2+^-depleted state was obtained by addition of 50 mM EDTA, and the Zn^2+^-saturated state was obtained with the addition of 8 μM and 2 mM ZnCl_2_ for LuZi and BLZinCh-Pro, respectively. Measurements were performed with 10 nM sensor protein and 1000-fold diluted NLuc substrate in 50 mM HEPES (pH 7.1) at 22 °C. Bars represent average values ± standard deviation (s.d.).

For LuZi-1, the emission ratio obtained in 2% serum was similar to that obtained in the presence of excess Zn^2+^, showing that this sensor was fully saturated (Figure 4A). Thus, the high affinity of LuZi-1 renders it unsuitable for free Zn^2+^ detection in serum. In contrast, LuZi-4.2 displayed an emission ratio between that of the Zn^2+^-depleted and Zn^2+^-saturated state, corresponding to 80% of the sensor being bound to Zn^2+^, which translates into a free Zn^2+^ concentration of 1.2 ± 0.7 nM. Unfortunately, while the affinity of LuZi-6.2 is in the proper affinity range, the small difference in the emission ratio observed between the Zn^2+^-free and Zn^2+^-bound states prevented reliable determination of the free Zn^2+^ concentration with this sensor. Furthermore, all three LuZi sensors displayed a smaller maximal change in the emission ratio in serum, mainly due to a higher emission ratio for the Zn^2+^-depleted state in serum compared to buffer. This higher emission ratio may be due to interactions of serum proteins that subtly affect the equilibrium between the putative high and low BRET Zn^2+^-bound states.

As the LuZi-4.2 sensor showed an attenuated change in the emission ratio in serum, we next explored the performance of the BLZinCh-Pro sensors to measure the serum free Zn^2+^ concentration. BLZinCh-P20, -P30, -P40, and -P50 all displayed a large change in the emission ratio, similar to the results in buffer (Figure 3A). Furthermore, the sensors yielded free Zn^2+^ concentrations of respectively 4.8 ± 0.6 nM, 3.8 ± 0.2 nM, 3.0 ± 0.2 nM, and 2.8 ± 0.2 nM (Figure 4B). It should be noted that the value obtained for BLZinCh-P20 is less reliable, because the sensor is almost fully saturated. The free Zn^2+^ concentrations obtained with LuZi 4.2 and BLZinCh-P30, -P40, and -P50 are similar to those previously reported using ZnAF-2 (1–3 nM) but approximately 10-fold higher than those reported using ZinPyr-1.^19^ This difference might be due to undesired interaction of the Zinpyr-1 sensor with serum components or because the affinities of the BLZinCh-Pro and LuZi sensors in serum differ slightly from those determined in buffer. The latter is less likely because the LuZi and BLZinCh-Pro sensors employ different Zn^2+^-binding domains. Irrespective of the exact number, the LuZi and BLZinCh-Pro sensors provide easy-to-use new tools for the quantification of free Zn^2+^ in serum and show great promise to be utilized in a clinical setting to detect zinc deficiencies.

### Bioluminescent Quantification of Intracellular Cytosolic Free Zn^2+^

**Figure 5:**
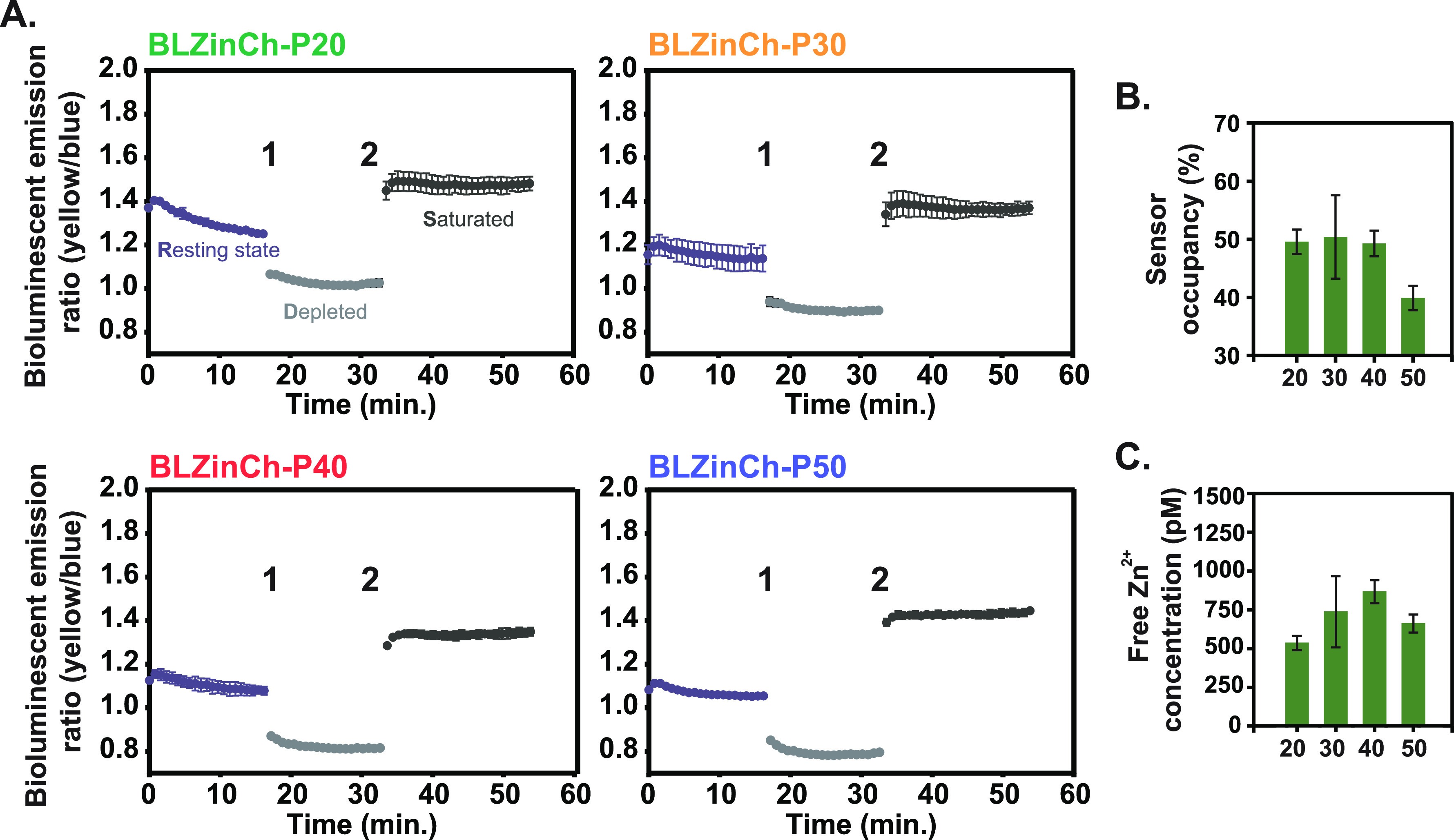
Monitoring changes in cytosolic free Zn^2+^ in HeLa cells expressing BLZinCh-Pro sensors. (A) Bioluminescence emission ratio (emission 505–545 nm/emission 400–455 nm) of HeLa cells expressing the BLZinCh-Pro sensor proteins in a resting state and after subsequent addition of 50 μM TPEN (1), representing the depleted state, and 350 μM Zn^2+^ with 10 μM pyrithione (2), inducing the saturated state. Bioluminescence of the cell suspension was monitored on a plate reader using the 3000-fold diluted NLuc substrate in 20 mM HEPES (pH 7.4), 140 mM NaCl, 2.5 mM KCl, 1.8 mM CaCl_2_, and 1.0 mM MgCl_2_, at 37 °C. Measurements were paused for ∼1 min during the additions at time points 1 and 2. Data points show the average of three measurements, and the error bars represent the standard deviation (s.d.). (B) Sensor occupancy (calculated using eq 4 in the Methods section) of the BLZinCh-Pro variants. (C) Intracellular free Zn^2+^ concentrations measured with the different BLZinCh-Pro sensors (calculated using eq 1). Bars represent average values ± standard deviation (s.d.).

An advantage of using BRET-based detection over FRET is the ability to use a standard plate reader to measure intracellular free Zn^2+^ concentrations in a population of cells. We previously applied the BLZinCh-1 sensor to measure cytosolic free Zn^2+^ levels in HeLa cells. However, the affinity and limited change in the emission ratio of BLZinCh-1 were not optimal to measure fluctuations in free Zn^2+^.^27^ To characterize the intracellular performance of the BLZinCh-Pro variants, we transiently transfected HeLa cells with each sensor variant and used the transfected cells to determine the cytosolic free Zn^2+^ levels with a plate reader. For each BLZinCh-Pro sensor, a Zn^2+^ concentration dependent change in the emission ratio was observed, with a decrease in the emission ratio upon Zn^2+^-depletion after addition of the chelator TPEN (time point 1 in Figure 5A) and an increase in the emission ratio upon addition of saturating amounts of Zn^2+^ in the presence of the ionophore pyrithione (time point 2 in Figure 5A). In addition to confirming the successful expression of functional sensor proteins, these results also show that the response to fluctuations in cytosolic free Zn^2+^ is rapid and complete within a few minutes. The BLZinCh-Pro sensors show a sensor occupancy between 40 and 50%, which is optimal to measure both increases and decreases in free Zn^2+^ (Figure 5B). Not surprisingly given the relatively small differences in affinity between the sensors, similar sensor occupancies were observed, although the sensor occupancy of BLZinCh-P50 was indeed found to be the lowest. Based on the sensor occupancy and the experimentally determined *K*_D_ for Zn^2+^ binding, the concentration of cytosolic free Zn^2+^ could be calculated (eq 1), yielding values of 536 ± 46 pM, 738 ± 231 pM, 867 ± 75 pM, and 661 ± 59 pM for the experiments with BLZinCh-P20, -P30, -P40, and -P50, respectively (Figure 5C). These values are in agreement with the 0.5–1 nM free Zn^2+^ that is typically observed for the concentration of free Zn^2+^ in the cytosol of mammalian cells using fluorescence-based sensors and probes.^21,40,41^ Note that the BLZinCh-Pro sensors are also expected to be attractive FRET sensors for live cell imaging using fluorescence microscopy. In comparison with the BLZinCh-1 and eZinCh-2 sensors, the BLZinCh-Pro sensors have an affinity that is better tuned to the concentration of free Zn^2+^ in the cytosol, and they also display a larger change in the emission ratio between the Zn^2+^-free and Zn^2+^-bound states (700% change for BLZinCh-P40 compared to 400% for BLZinCh-1, Figure S4).

## Conclusions

In conclusion, two new bioluminescent sensor platforms were developed that allow fast, robust, and sensitive quantification of free Zn^2+^ in serum and in the cytosol of mammalian cells. The LuZi sensors, based on mutually exclusive split NLuc complementation and BRET to a red fluorescent Cy3 dye, are modular by design and yield a robust change in red to blue emission upon binding to Zn^2+^. For the second platform, we substituted the long flexible GGS linker in the previously reported BLZinCh-1 sensor with rigid polyproline linkers yielding four different sensor proteins with a 3–4-fold improved change in the emission ratio and Zn^2+^ affinities between 543 and 992 pM that are optimal for measuring cytosolic free Zn^2+^ concentrations. Measurements with the BLZinCh-Pro sensors and the LuZi-4.2 sensor revealed similar values for the concentration of free Zn^2+^ in (diluted) serum of 1–3 nM. The dual readout BRET/FRET BLZinCh-Pro sensors were also shown to be ideal for intracellular free Zn^2+^ measurements, providing an attractive alternative for more complex and expensive methods based on live cell imaging using fluorescence microscopy or FACS. The metal selectivities of the LuZi and BLZinCh-Pro sensors will be the same as those of their parent sensor proteins, eCALWY and eZinCh2, respectively. Previous work on the eCALWY sensors showed interference by Cu^+^, Pb^2+^, and Cd^2+^, but no interference by physiologically relevant concentrations of Ca^2+^, Mg^2+^, Fe^2+^, Ni^2+^, and Cu^2+^. Moreover, mutation of one of the four coordinating cysteine residues in eCALWY4-6 also abrogated Cu^+^ binding.^22,24^ The metal specificity of eZinCh-2 also showed interference by soft bivalent ions such as Pb^2+^ and Cd^2+^, but no interference by physiologically relevant metal ions such as Ca^2+^, Mg^2+^, Fe^2+^, Co^2+^, and Cu^2+^.^23^

An important aspect in the optimization of both platforms was tuning the length and stiffness of the linkers between the individual domains in the sensors. The optimized polyproline linkers in the BLZinCh-Pro platform are more rigid compared to the original flexible GGS linker, increasing the effective distance between the fluorescent proteins in the Zn^2+^-depleted state of the sensor and attenuating the affinity for Zn^2+^ by decreasing the effective concentration for complex formation. The effect of linker optimization can also be more complex. Shortening the linker between the metal-binding domains and the LB and SB2 domains in the LuZi sensors improved the change in the emission ratio for LuZi-4, while the sensor properties of LuZi-6 remained unaltered. This difference can be understood by considering the equilibrium between two Zn^2+^-bound states, one state in which SB2 still interacts with the LB domain (high BRET) and one state in which the interaction between SB2 and LB is disrupted and LB binds to SB1 (low BRET; Figure 2C). The equilibrium between these two states is determined by the relative effective concentrations of the different domains in each complex, which in turn is determined by linker lengths and the distances that linkers need to bridge. In the low BRET, Zn^2+^-bound state, the linker between the Atox1 and WD4 domains needs to span a distance of 50 Å. We have previously shown that the linker used in the LuZi-4 sensor (containing 18 GGS repeats) is more favorable in this low BRET state than the linker used in the LuZi-6 sensor (6 GGS repeats), translating into an at least 10-fold higher effective concentration for complex formation.^42^ However, if the distance that this linker needs to bridge is smaller in the high-BRET, Zn^2+^-bound state, this state will become relatively more favorable for the sensor with a shorter linker.^32^ These effects are likely to be subtle and also be determined by the relative stability of Zn^2+^ binding in each of the two conformational states. It is important to emphasize that while the existence of two different Zn^2+^-bound conformational states provides a model for explaining the observed effects of linker lengths on sensor properties, additional biophysical studies are required to provide more direct evidence for these two conformational states.

The LuZi-4.2, BLZinCh-P30, BLZinCh-P40, and BLZinCh-P50 sensors provide attractive tools for measuring free Zn^2+^ concentrations in blood plasma and serum. Compared to fluorescence-based sensors, these bioluminescent sensors do not require external illumination, making them well suited for integration in cheap and easy-to-use paper-based devices or microfluidic chips with a simple mobile phone as the readout method.^19,43,44^ Because the LuZi sensors are based on complementation of split NanoLuc, their brightness is approximately 10-fold lower than that of the BLZinCh-Pro sensors, which might become a disadvantage when measuring in very small volumes. At present, the affinities of the bioluminescent sensors are at the high end of the physiologically relevant range, which may make it challenging to reliably measure elevated concentrations of free Zn^2+^. Further decreasing the Zn^2+^ affinities to a *K*_D_ of ∼5 nM would thus be of interest. This might be achieved by a comprehensive screening of linker lengths and linker rigidity to increase the dynamic range of the LuZi-6 sensor (see above) or by further attenuating the Zn^2+^ affinity of the BLZinCh-Pro sensors by more direct fusion of the rigid proline linkers to the Cerulean and Citrine domains. The link between free Zn^2+^ and the overall Zn^2+^ status remains unclear. By providing an easy-to-use and cheap method to measure free Zn^2+^ concentrations, these sensors provide an opportunity to establish whether the concentration of free Zn^2+^ is a better biomarker for zinc nutritional status than the total zinc concentration currently determined using AAS or ICP-MS. In addition, the bioluminescent sensors reported here also represent attractive tools to determine free Zn^2+^ concentrations in other complex media including saliva, cerebrospinal fluid, and milk.

## Methods

### Expression and Purification of Sensor Proteins

The construction of the expression plasmids is described in the Supporting Information. The plasmids encoding the LuZi sensors were cotransformed into *E. coli* BL21 (DE3) competent bacteria (Novagen) together with the pEVOL plasmid encoding the tRNA/tRNA synthetase pair for the incorporation of the unnatural amino acid pAzF. The pEVOL vector was a gift from Peter Schultz (Addgene plasmid #31186).^45^ Single colonies were picked and used to inoculate 8 mL 2YT medium cultures supplemented with 30 μg/mL kanamycin and 25 μg/mL chloramphenicol, which were grown overnight at 37 °C and 250 rpm. Subsequently, the cultures were transferred into 1 L 2YT cultures containing 30 μg/mL kanamycin and 25 μg/mL chloramphenicol and were grown at 37 °C and 160 rpm until an OD_600_ of 0.6. Expression was induced using 0.1 mM isopropyl β-d-1-thiogalactopyranoside (IPTG) and 0.2% arabinose in the presence of 1 mM pAzF (Bachem, F-3075.0001), and the induced cultures were further grown overnight at 18 °C and 160 rpm. The bacteria were harvested and lysed using the BugBuster reagent (Novagen) supplemented with Benzonase (Novagen). The obtained sensor proteins were purified using Ni-NTA affinity chromatography followed by Strep-Tactin purification according to the manufacturer’s instructions. The protein purity was confirmed by SDS-PAGE (Figure S1), and the proteins were stored at −80 °C until further use.

The pET28a plasmids encoding the BLZinCh-Pro sensors were transformed into *E. coli* BL21 (DE3) competent bacteria. Single colonies were picked and used to inoculate 8 mL LB medium cultures supplemented with 30 μg/mL kanamycin, which were grown overnight at 37 °C and 250 rpm. Subsequently, the cultures were transferred into 1 L LB cultures containing 30 μg/mL kanamycin and were grown at 37 °C and 160 rpm until an OD_600_ of 0.6. Expression was induced using 0.5 mM IPTG, and the induced cultures were further grown overnight at 18 °C and 160 rpm. The sensor proteins were purified as described above; however, 1 mM tris(2-carboxyethyl)phosphine (TCEP, Sigma-Aldrich) was supplemented to the lysis reagent and to all wash and elution buffers. In addition, 50 μM DL-dithiothreitol (DTT, Sigma-Aldrich) was added to the Strep-Tactin elution buffer (Figure S2). In the end, the Strep-Tactin elution was concentrated and exchanged with 150 mM 4-(2-hydroxyethyl)-1-piperazineethanesulfonic acid (HEPES, pH 7.1), 100 mM NaCl, 10% (v/v) glycerol, 50 μM DTT, and 1 mM TCEP using Amicon Ultra-4 Centrifugal Filter Units (molecular weight cutoff 10 kDa, Millipore).

### Fluorophore Labeling

The purified LuZi sensor proteins were conjugated with DBCO-Sulfo-Cy3 (Lumiprobe, 113F0) in a 30-fold molar excess overnight at RT. Subsequently, the excess dye was removed using an Amicon Ultra-4 centrifugal filter (molecular weight cutoff 10 kDa; Millipore). The dye-to-protein ratio was calculated using eq 2. The absorbance (*A*) of the protein and dye was measured at 280 and 555 nm, respectively, using the UV–vis mode of NanoDrop 1000 with a path length (*L*) of 0.1 cm. The extinction coefficients (ε) of 32,890 M^–1^ cm^–1^ and 151,000 M^–1^ cm^–1^ for the protein and dye, respectively, were determined using the ProtParam tool of ExPASy. A correction factor (CF) of 0.06 was used to correct for the absorbance of the dye at 280 nm.
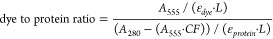
2

### Sensor Characterization

Bioluminescence emission spectra were measured in a white flat-bottom 384-well plate (Nunc, Thermo Scientific) using the Tecan Spark plate reader and an integration time of 100 ms. Measurements were carried out in a 20 μL volume with the 2 nM sensor protein for the LuZi sensor proteins and 0.2 nM for the BLZinCh-Pro sensor proteins in a buffer consisting of 150 mM HEPES (pH 7.1), 100 mM NaCl, 10% (v/v) glycerol, 5 μM DTT, and 1 mM TCEP. Different concentrations of ZnCl_2_ were added together with different Zn^2+^ chelators depending on the concentration range measured. One mM ethylenediaminetetraacetic acid (EDTA) was used to measure in the low picomolar concentrations, 1 mM *N*-(2-hydroxyethyl)ethylenediamine-*N*,*N*′,*N*′-triacetic acid (HEDTA) was used for higher picomolar concentrations, and 1 mM 1,3-diamino-2-hydroxypropane-*N*,*N*,*N*′,*N*′-tetraacetic acid (DHPTA) was used to measure in the high picomolar range. One mM ethylene glycol-bis(2-aminoethyl ether)-*N*,*N*,*N*′,*N*′-tetraacetic acid (EGTA) was used to measure in the nanomolar range. All chelators were obtained from Sigma-Aldrich. The free Zn^2+^ concentrations for each chelator and a certain ZnCl_2_ concentration were calculated with the program MaxChelator using the stability constants present within the program, as previously described.^22^ The mixed samples were incubated for 15–20 min, followed by the addition of the 1000-fold diluted NLuc substrate (Promega, N1110) for the LuZi sensors and the 3200-fold diluted NLuc substrate for the BLZinCh-Pro sensors. The *K*_D*,app*_ was determined by fitting the bioluminescence emission ratio using eq 3. [Zn^2+^] represents the calculated free Zn^2+^ concentration, P1 is the difference between the ratio in the Zn^2+^-saturated and Zn^2+^-depleted state, and P2 is the emission ratio in the Zn^2+^-depleted state.
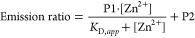
3

### Serum Measurements

The measurements in serum (Sigma-Aldrich, H4522) were performed in a white flat-bottom 384-well plate with a sample volume of 20 μL using the 10 nM sensor and 1000× diluted NLuc substrate. Serum was 50 times diluted with 50 mM HEPES (pH 7.1). The Zn^2+^-depleted state was created by adding 50 mM EDTA, and the Zn^2+^-saturated state was created by addition of 8 μM ZnCl_2_ for the LuZi sensors and 2 mM ZnCl_2_ for the BLZinCh-Pro sensors. All samples were prepared in triplicate, and the luminescence was measured directly after adding the substrate, using the Tecan Spark plate reader. The free Zn^2+^ concentration was calculated using eq 1.

### Cytosolic Free Zn^2+^ Measurements

Cell culture, transfection, and bioluminescent measurements were executed as described in ref (30). In short, HeLa cells were grown in Dulbecco’s Modified Eagle Medium (DMEM) supplemented with 4.5 g/L d-glucose, 0.58 g/L l-glutamine, 10% fetal bovine serum (FBS), 100 U/mL penicillin, and 100 μg/mL streptomycin (all from Life Technologies) at 37 °C and 5% CO_2_ in Falcon corning T75 culture flasks (REF 353136). One day before transfection, 140 000 cells were seeded in a six-well plate (Corning). When cells reached ∼80% confluency, the cells were transfected with 2 μg of pCMV-BLZinCh-1, -P20, -P30, -P40, or -P50 and 3 μL of Lipofectamine 3000 (Life Technologies) in Opti-MEM Reduced Serum Medium (Life Technologies). After 6 h, the medium was refreshed with DMEM. Two days after transfection, trypsin (Thermo Fisher Scientific) was used to release the cells from the wells, and the cells were subsequently resuspended in 1 mL of imaging buffer (20 mM HEPES (pH 7.4), 140 mM NaCl, 2.5 mM KCl, 1.8 mM CaCl_2_, and 1.0 mM MgCl_2_). Fifteen microliters of the HeLa cells was transferred to a 96-well plate (Nunc, Thermo Scientific) to which buffer was added to make a final volume of 150 μL. The NLuc substrate in a final 3000-fold dilution was added, and bioluminescence was monitored on a Tecan Spark 10 M plate reader using filters for the detection of NLuc-Cerulean (400–455 nm) and Citrine (500–545 nm) emission. Integration times of 1 s were used, and the temperature was set at 37 °C. The measurement was performed for 50 min, during which 50 μM *N*,*N*,*N*′,*N*′-tetrakis(2-pyridylmethyl)ethylenediamine (TPEN, Sigma-Aldrich) was added to the cells after 17 min, and 350 μM ZnCl_2_ and 10 μM 2-mercaptopyridine *N*-oxide (pyrithione, Acros Organics) were added after 33 min. Sensor occupancies were calculated using eq 4, and the Zn^2+^ levels were calculated using eq 1. Rmin and Rmax represent the steady-state emission ratios after the addition of TPEN and Zn^2+^/pyrithione, respectively, and *R* is the steady-state emission ratio of the cells in the resting state.
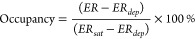
4

## Supporting Information Available

Additional experimental methods, SDS-PAGE analysis of sensor proteins, fluorescence emission spectra, labeling efficiencies, bioluminescent measurements in serum, and DNA sequences of sensor proteins (PDF)
