# Supplementary material for: Ratiometric Bioluminescent Zinc Sensor Proteins to Quantify Serum and Intracellular Free Zn2+
Source: ACS Chem Biol. 2022 May 25;17(6):1567–76. doi: 10.1021/acschembio.2c00227 (PMC9207811; doi:10.1021/acschembio.2c00227)
Supplement: Supplementary file 1 — cb2c00227_si_001.pdf [file cb2c00227_si_001.pdf]

Supporting information for

Ratiometric bioluminescent zinc sensor proteins to quantify serum and intracellular free Zn^{2+}

Claire M. S. Michielsen[||,†,‡], Eva A. van Aalen[||,†,‡] and Maarten Merkx[†,‡,*]

[†]Laboratory of Chemical Biology, Department of Biomedical Engineering and [‡]Institute for Complex Molecular Systems, Eindhoven University of Technology, P.O Box 513, 5600 MB Eindhoven, The Netherlands.
*Email: m.merkx@tue.nl

Content

Content		Page
Supplementary Methods	Construction expression plasmids	S2
Table S1	Primers used for cloning of the sensor protein plasmids	S3
Table S2	$K_{D,app}$ values of the protein sensors	S3
Figure S1	SDS-PAGE analysis of the purified LuZi sensor proteins	S4
Figure S2	SDS-PAGE analysis of the BLZinCh-Pro sensor proteins	S5
Figure S3	Agarose gel analysis of proline linker lengths	S6
Figure S4	Fluorescence emission spectra of BLZinCh-P40	S6
Figure S5	Bioluminescence emission ratio of LuZi-4 in 10% serum	S7
Figure S6	Nucleotide and amino acid sequence of 50x proline linker	S8
Figure S7	Nucleotide and amino acid sequence of BLZinCh-1	S8-9
Figure S8	Nucleotide and amino acid sequence of LuZi-4	S10-11

Supplementary Methods. Construction of expression plasmids

Plasmids pET28a-BLZinCh-1, pET28a-CTX-NB-LUMABS and pET28a-eCALWY-1, -4, -6 were available from previous work in our lab.[24,30,33] The restriction enzymes KpnI-HF and SpeI-HF were used to digest the CTX-NB-LUMABS vector and the digested backbone was isolated using the QIAquick Gel Extraction Kit. Overhang PCR primers CALWY-KpnI.FOR and CALWY-SpeI.REV (Supplementary Table 1) were designed to isolate a linear construct containing the Zn^{2+}-binding domains from the eCALWY plasmids with the restriction site KpnI in front of the Atox1 domain and SpeI after the WD4 domain. The PCR products were purified using the QIAquick PCR Clean-Up Kit and subsequently digested using KpnI-HF and SpeI-HF. Again the QIAquick PCR Clean-Up Kit was used to purify the digested insert. Next, the inserts containing the Zn^{2+}-binding domains were ligated into the digested backbone of the CTX-NB-LUMABS vector to form the LuZi plasmids, which was confirmed with Sanger sequencing (Figure S9). Furthermore, to develop the optimized LuZi-4.2 and LuZi-6.2, the linkers fusing Atox1 to LB and WD4 to SB2 were shortened using overhang PCR. The primer LBPstI.FOR was used together with LBKpnI.REV to obtain a construct with at the 5' end the restriction site PstI, which is located in LB, and at the 3' end the KpnI restriction site, which is located at the end of the new LB-Atox1 linker. In parallel, the primer SB2Link1.FOR was used together with XhoI.REV to obtain a construct with at the 5' end the restriction site SpeI and the new WD4-SB2 linker and at the 3' end the restriction site XhoI, which is located after the stop codon at the end of the LuZi sequence (Supplementary Table 1, Figure S9). The PCR constructs were digested using PstI-HF and KpnI-HF for the LB-Atox1 construct and SpeI-HF and XhoI-HF for the WD4-SB2 construct. The digestion mixtures were purified using the QIAquick PCR Clean-Up Kit. The original LuZi plasmid was first digested using the restriction sites SpeI-HF and XhoI-HF. The digested backbone was isolated using the QIAquick Gel Extraction Kit. Subsequently, the digested WD4-SB2 insert was ligated into the digested LuZi plasmid. Next, the previous obtained LuZi plasmid containing the new WD4-SB2 linker was again digested but now using PstI-HF and KpnI-HF. The digested backbone was isolated with the QIAquick Gel Extraction Kit and the digested LB-Atox1 insert was ligated into the backbone, resulting in the optimized LuZi plasmid containing both new linkers. All cloning steps were confirmed with Sanger sequencing.

To develop the pET28-BLZinCh-Pro expression plasmids, the two BseRI restriction sites present in the pET28-BLZinCh-1 plasmids were first mutated out using the QuikChange Multi Site-Directed Mutagenesis Kit and primer mutBseRI-BLZinCh.FOR (Supplementary Table 1). Subsequently, the pUC57 vector containing the designed 50x-proline linker (Figure S7), ordered from GenScript, and the BLZinCh-1 vector were digested using SacI-HF and KpnI-HF. The digested BLZinCh-1 vector backbone and the 50x proline linker insert were isolated using the QIAquick Gel Extraction Kit. The isolated insert and backbone were ligated using the T4 ligase enzyme. Subsequently, the vectors of the other BLZinCh-Pro variants were obtained by partially digesting the BLZinCh-P50 vector with the BseRI enzyme and ligating the digested products. The ligated vectors were transformed in NovaBlue cells and grown overnight. To determine which BLZinCh-Pro variants were obtained (P20, P30 or P40), colony PCR was performed using primers Plink-BLZinCh.FOR and Plink-BLZinCh.REV (Supplementary Table 1) to screen the DNA of the colonies. A 3% agarose gel was used to distinguish the linker lengths of the colony PCR products, which differ 30 base pairs (Figure S3). The vectors of the colonies containing the desired linker length were isolated using the QIAprep Spin Miniprep Kit. The obtained sequences of BLZinCh-P50, BLZinCh-P40, BLZinCh-P30 and BLZinCh-P20 were confirmed by Sanger sequencing. Furthermore, all four BLZinCh-Pro variants were cloned into a pCMV vector for mammalian cell expression. pCMV-BLZinCh-1 from Stijn Aper and the pET28-BLZinCh-Pro plasmids were digested using restriction sites NheI and XhoI. After isolating the digested constructs using the QIAquick Gel Extraction Kit, the digested sensor sequences were ligated into the pCMV backbone using the T4 ligase enzyme. Construction of the pCMV-BLZinCh-Pro plasmids was confirmed by Sanger sequencing.

Table S1. Primers used for cloning of the sensor protein plasmids. Primers designed for the construction and optimization of the LuZi sensor and incorporation of the polyproline linker in BLZinCh-1.

Name primer	Sequence
CALWY-KpnI.FOR	5’ GCGGCGGGTACCATGCCGAAGCACGAGTTC
CALWY-SpeI.REV	5’ GCGGCGACTAGTGACTGAAGCCTCAAATCC
LBPstI.FOR	5'CCAGTTTGCTGCAGAATCTCG
LBKpnI.REV	5’ACATCTGGTACCGTTGATGGTTACTCGGAACAGC
SB2Link1.FOR	5’ACATCTACTAGTTAGGGCTCCGTTACCGGCTATCGTC
XhoI.REV	5’GTGGTGCTCGAGTGCGGC
mutBseRI-BLZinCh.FOR	5’GGTGAGCAAGGGCGAAGAGCTGTTCACCGG
Plink-BLZinCh.FOR	5’CAAGTCCGGAGGCGGCGAG
Plink-BLZinCh.REV	5’GCCCTTGCTCACCATGGGTACC
CMVpromoter.FOR	5'GGTGGGAGGTCTATATAAGC
SV40pA.REV	5'GAAATTTGTGATGCTATTGC

Table S2. $K_{D,app}$ values of the protein sensors. All dose-response assays to determine the $K_{D,app}$ values were performed in 150 mM HEPES (pH 7.1), 100 mM NaCl, 10% (v/v) glycerol, 5 µM DTT, 1 mM TCEP and 1 mg/mL BSA at 20 °C.

Sensor Protein	Sensor protein concentration	$K_{D,app}$
**LuZi-1**	2 nM	12.5 ± 1.0 pM
**LuZi-4**	2 nM	176 ± 32 pM
**LuZi-4.2**	2 nM	221 ± 18 pM
**LuZi-6**	2 nM	1.15 ± 0.1 nM
**LuZi-6.2**	2 nM	708 ± 147 pM
**BLZinCh-P20**	0.2 nM	543 ± 45 pM
**BLZinCh-P30**	0.2 nM	693 ± 57 pM
**BLZinCh-P40**	0.2 nM	889 ± 76 pM
**BLZinCh-P50**	0.2 nM	992 ± 80 pM

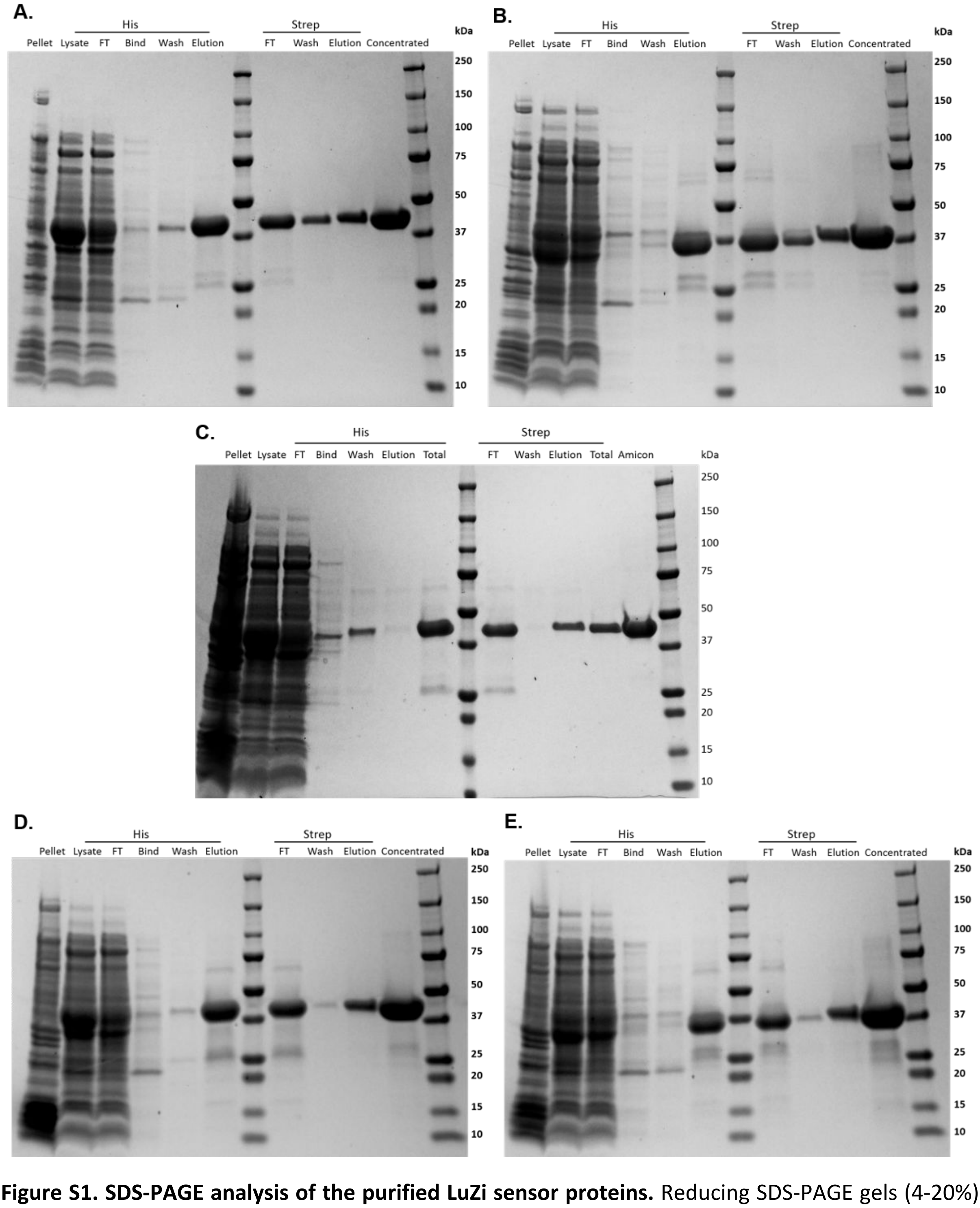

Figure S1. SDS-PAGE analysis of the purified LuZi sensor proteins. Reducing SDS-PAGE gels (4-20%) analysis of the expression (in *E. coli* BL21 (DE3)) and purification with Ni^{2+} affinity chromatography and Strep-Tactin chromatography of (A) LuZi-1, (B) LuZi-6, (C) LuZi-4, (D) LuZi-4.2 and (E) LuZi-6.2.

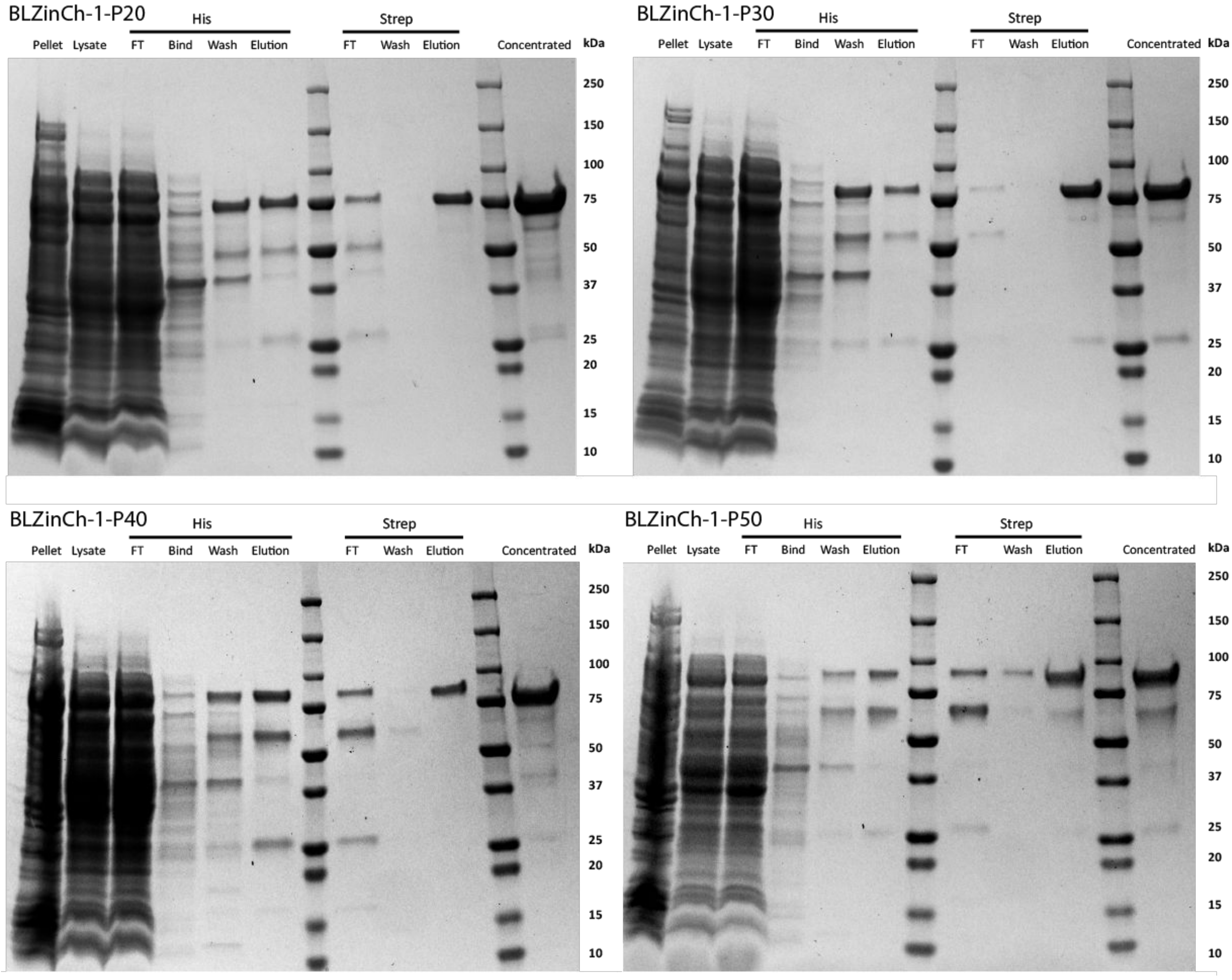

Figure S2. SDS-PAGE analysis of the BLZinCh-Pro sensor proteins. Reducing SDS-PAGE gels (4-20%) analysis of the of the four BLZinCh-Pro variants (BLZinCh-P20, BLZinCh-P30, BLZinCh-P40 and BLZinCh-P50). After expression of the proteins in *E. coli* BL21 (DE3), the BLZinCh-Pro sensors were purified using Ni^{2+} affinity chromatography and Strep-Tactin chromatography.

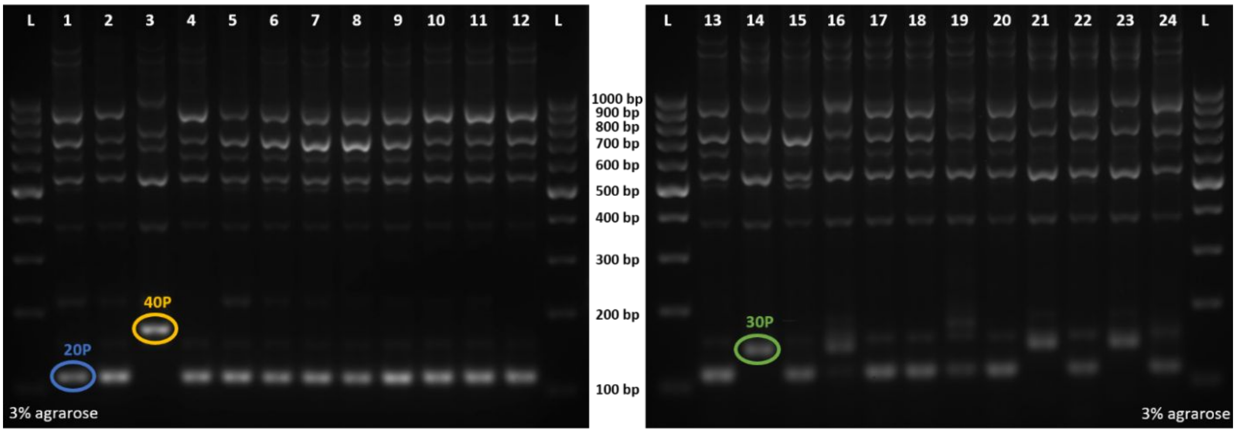

Figure S3. Agarose gel analysis of proline linker lengths. Development of DNA encoding for BLZinCh-P20, BLZinCh-P30 and BLZinCh-P40. BLZinCh-P50 was digested with the BseRI restriction enzyme and the digested products were subsequently ligated, generating sensors with different polyproline linkers. The 3% agarose gels of the colony PCR reaction, performed with primers Plink-BLZinCh.FOR and Plink-BLZinCh.REV (Supplementary Table 1), shows the successful development of DNA encoding for BLZinCh-P20, BLZinCh-P30 and BLZinCh-P40.

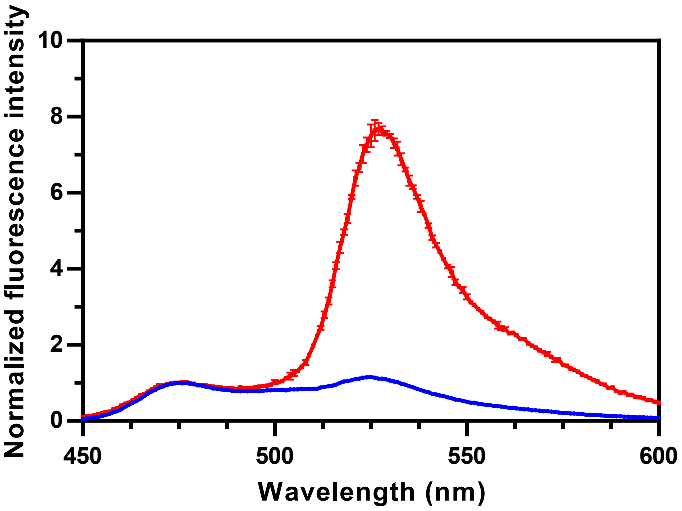

Figure S4. Fluorescence emission spectra of BLZinCh-P40. Fluorescence emission spectrum (excitation 400 nm) normalized to emission of Cerulean (475 nm) of BLZinCh-P40 in Zn^{2+}-depleted (blue) and Zn^{2+}-saturated state (red). The Zn^{2+}-depleted state was obtained using 1 mM DHPTA and the Zn^{2+}-saturated state was obtained using 1 mM NTA and 0.9 mM $ZnCl_2$. Measurements were performed using 1 µM BLZinCh-P40 in 150 mM HEPES (pH 7.1), 100 mM NaCl, 10% (v/v) glycerol, 5 µM DTT, 1 mM TCEP and 1 mg/mL BSA at 20 °C.

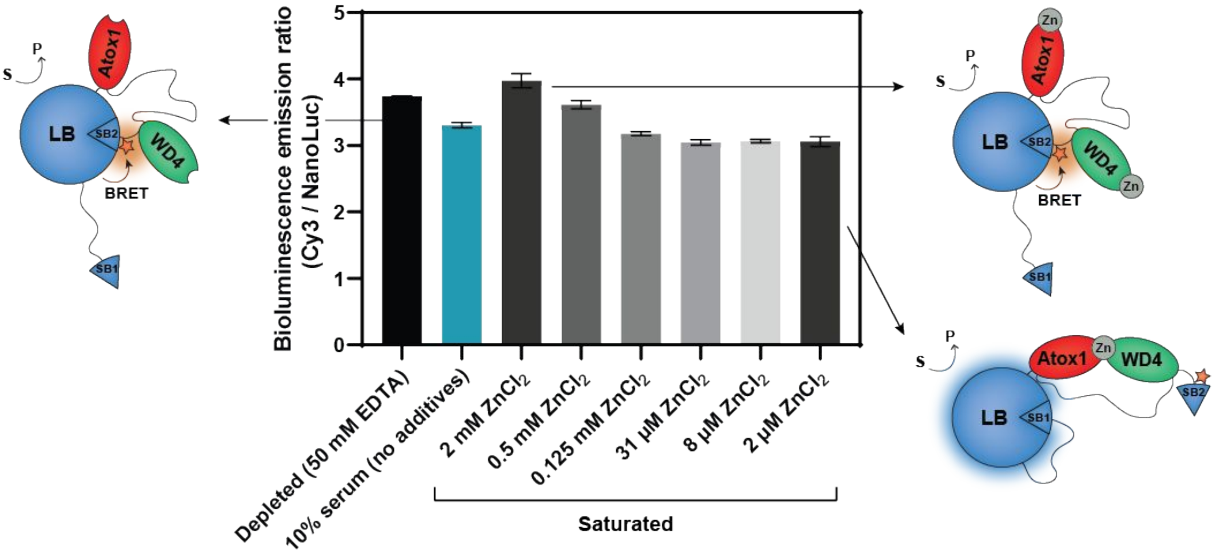

Figure S5. Bioluminescence emission ratio of LuZi-4 in 10% serum. Bioluminescence emission ratio (578 nm/458 nm) of LuZi-4.2 in 10% serum. The Zn^{2+}-depleted state was obtained by adding 50 mM EDTA and the Zn^{2+}-saturated state was obtained by adding different $ZnCl_2$ concentrations. Measurements were performed in triplicate using 10 nM LuZi-4.2 and 1000-fold diluted NLuc substrate in 50 mM HEPES (pH 6.5).

BLZinCh-proline-linker_pUC57

```
agcggcgagctcccacctccaccgccgcctccacctccaccgccacccctccaccccg
 S  G  E  L  P  P  P  P  P  P  P  P  P  P  P  P  P  P  P  P
ccacctcctccaccgccaccaccgcccccgccacctcctccacctccccaccgcccct
 P  P  P  P  P  P  P  P  P  P  P  P  P  P  P  P  P  P  P  P
ccacctcctccaccgcccccaccgcccccgccacctcctccgcggggctcggtacccggc
 P  P  P  P  P  P  P  P  P  P  P  P  P  P  R  G  S  V  P  G
agc
 S
```

(SacI site **gagctc** and KpnI site **ggtacc** shown in bold on the page.)

Figure S6. Nucleotide and amino acid sequence of 50x proline linker. Nucleotide and amino acid sequence of the 50x polyproline linker for the replacement of the flexible GGS-linker in the BLZinCh-1 protein sensor. The SacI and KpnI restriction sites used for cloning are shown in bold.

BLZinCh-1_pET28a(+)

```
atgggcagcagccatcatcatcatcatcacagcagcggcctggtgctagccatggtattt
 M  G  S  S  H  H  H  H  H  H  S  S  G  L  V  L  A  M  V  F
Actcttgaagattttgtcggtgattggcgccagaccgccggctataacctggaccaagtg
 T  L  E  D  F  V  G  D  W  R  Q  T  A  G  Y  N  L  D  Q  V
cttgaacagggcggggttagcagcctgtttcaaaacctgggggtgagtgtcacgccaatt
 L  E  Q  G  G  V  S  S  L  F  Q  N  L  G  V  S  V  T  P  I
cagcgcatcgttctgtcgggagagaatggtctgaaaatcgatatccacgtcattatcccg
 Q  R  I  V  L  S  G  E  N  G  L  K  I  D  I  H  V  I  I  P
tacgaaggtctttctggtgatcagatggggcagatagaaaaaatattcaaagtggtgtac
 Y  E  G  L  S  G  D  Q  M  G  Q  I  E  K  I  F  K  V  V  Y
ccagtagacgatcatcacttcaaggttatactgcactatggcaccctcgttatcgatggc
 P  V  D  D  H  H  F  K  V  I  L  H  Y  G  T  L  V  I  D  G
gttactccgaatatgatcgattactttggcgtccttatgaaggtattgcggtgttcgac
 V  T  P  N  M  I  D  Y  F  G  R  P  Y  E  G  I  A  V  F  D
ggtaaaaaaattacggttaccgggacgctctggaatggtaataaaatcattgatgagcgc
 G  K  K  I  T  V  T  G  T  L  W  N  G  N  K  I  I  D  E  R
ttgataaacccagatggcagccttctgttcagagttacgataaacggggttacgggttgg
 L  I  N  P  D  G  S  L  L  F  R  V  T  I  N  G  V  T  G  W
cgactgtgcgaaagacatatggtgagcaagggcgaggagctgttcaccggggtggtgccc
 R  L  C  E  R  H  M  V  S  K  G  E  E  L  F  T  G  V  V  P
atcctggtcgagctggacggcgacgtaaacggccacaagttcagcgtgtccggcgagggc
 I  L  V  E  L  D  G  D  V  N  G  H  K  F  S  V  S  G  E  G
gagggcgatgccacctacggcaagctgaccctgaagttcatctgcaccaccggtaagctg
 E  G  D  A  T  Y  G  K  L  T  L  K  F  I  C  T  T  G  K  L
cccgtgccctggcccaccctcgtgaccaccctgacctggggcgtgcagtgcttcgcccgc
 P  V  P  W  P  T  L  V  T  T  L  T  W  G  V  Q  C  F  A  R
taccccgaccacatgaagcagcacgacttcttcaagtccgccatgcccgaaggctacgtc
 Y  P  D  H  M  K  Q  H  D  F  F  K  S  A  M  P  E  G  Y  V
caggagcgcaccatcttcttcaaggacgacggcaactacaagacccgcgccgaggtgaag
 Q  E  R  T  I  F  F  K  D  D  G  N  Y  K  T  R  A  E  V  K
ttcgagggcgacaccctggtgaaccgcatcgagctgaagggcatcgacttcaaggaggac
 F  E  G  D  T  L  V  N  R  I  E  L  K  G  I  D  F  K  E  D
ggcaacatcctggggcacaagctggagtacaacgccatcagcgacaacgtctatatcacc
 G  N  I  L  G  H  K  L  E  Y  N  A  I  S  D  N  V  Y  I  T
gccgacaagcagaagaacggcatcaaggccaacttcaagatccgccacaacatcgaggac
 A  D  K  Q  K  N  G  I  K  A  N  F  K  I  R  H  N  I  E  D
ggcagcgtgcagctcgccgaccactaccagcagaacacccccatcggcgacggccccgtg
 G  S  V  Q  L  A  D  H  Y  Q  Q  N  T  P  I  G  D  G  P  V
```

```
ctgctgcccgacaaccactacctgagcacccagtcccacctgtgcaaagaccccaacgag
 L  L  P  D  N  H  Y  L  S  T  Q  S  H  L  C  K  D  P  N  E
aagcgcgatcacatggtcctgctggagttcgtgaccgccgccgggatcactctcggcatg
 K  R  D  H  M  V  L  L  E  F  V  T  A  A  G  I  T  L  G  M
gacgagctgtacaagtccggaggcggcgagctcattcgtggcggatccggcggaagcggc
 D  E  L  Y  K  S  G  G  G  E  L  I  R  G  G  S  G  G  S  G
ggatccggcggtagcggcggatccggcggctccggcggatccggcggcagcggcggatcc
 G  S  G  G  S  G  G  S  G  G  S  G  G  S  G  G  S  G  G  S
ggtggaagcggtggatccggtggtagcggtggatccggtggaagcggtggatccggtggt
 G  G  S  G  G  S  G  G  S  G  G  S  G  G  S  G  G  S  G  G
agcggtggatccggggtccgcggggctcggtacccatggtgagcaagggcgaggagctg
 S  G  G  S  G  G  P  R  G  S  V  P  M  V  S  K  G  E  E  L
ttcaccggggtggtgcccatcctggtcgagctggacggcgacgtaaacggccacaagttc
 F  T  G  V  V  P  I  L  V  E  L  D  G  D  V  N  G  H  K  F
agcgtgtccggcgagggcgagggcgatgccacctacggcaagctgaccctgaagttcatc
 S  V  S  G  E  G  E  G  D  A  T  Y  G  K  L  T  L  K  F  I
tgcaccaccggcaagctgcccgtgccctggcccaccctcgtgaccaccttcggctacggc
 C  T  T  G  K  L  P  V  P  W  P  T  L  V  T  T  F  G  Y  G
ctgatgtgcttcgcccgctaccccgaccacatgaagcagcacgacttcttcaagtccgcc
 L  M  C  F  A  R  Y  P  D  H  M  K  Q  H  D  F  F  K  S  A
atgcccgaaggctacgtccaggagcgcaccatcttcttcaaggacgacggcaactacaag
 M  P  E  G  Y  V  Q  E  R  T  I  F  F  K  D  D  G  N  Y  K
acccgcgccgaggtgaagttcgagggcgacaccctggtgaaccgcatcgagctgaagggc
 T  R  A  E  V  K  F  E  G  D  T  L  V  N  R  I  E  L  K  G
atcgacttcaaggaggacggcaacatcctggggcacaagcttgagtacaactacaacagc
 I  D  F  K  E  D  G  N  I  L  G  H  K  L  E  Y  N  Y  N  S
cacaacgtctatatcatggccgacaagcagaagaacggcatcaaggtgaacttcaagatc
 H  N  V  Y  I  M  A  D  K  Q  K  N  G  I  K  V  N  F  K  I
cgccacaacatcgaggacggcagcgtgcagctcgccgaccactaccagcagaacaccccc
 R  H  N  I  E  D  G  S  V  Q  L  A  D  H  Y  Q  Q  N  T  P
atcggcgacggccccgtgctgctgcccgacaaccactacctgagctaccagtcccacctg
 I  G  D  G  P  V  L  L  P  D  N  H  Y  L  S  Y  Q  S  H  L
tgcaaagaccccaacgagaagcgcgatcacatggtcctgctggagttcgtgaccgccgcc
 C  K  D  P  N  E  K  R  D  H  M  V  L  L  E  F  V  T  A  A
gggatcactctcggcatggacgagctgtacaagtacaagcggccgcactcgagcaccacc
 G  I  T  L  G  M  D  E  L  Y  K  Y  K  R  P  H  S  S  T  T
accaccaccactgagatccggctgcactggtctcatcctcaatttgaaaaataa
 T  T  T  T  E  I  R  L  H  W  S  H  P  Q  F  E  K  *
```

Figure S7. Nucleotide and amino acid sequence of BLZinCh-1. Nucleotide and amino acid sequence of BLZinCh-1 in the pET28a(+) vector. The His-tag and Strep-tag are colored in grey and purple, respectively. NanoLuc, Cerulean and Citrine are depicted in dark blue, light blue and yellow, respectively. The BseRI restriction sites, which were removed before the incorporation of the polyproline linker to form the BLZinCh-1-Polyproline variants are shown in bold (light blue and yellow). The SacI and KpnI restriction sites used for cloning are shown in bold (black) and the flexible GGS-linker that is replaced by the polyproline linker to form the BLZinCh-1-Polyproline variants is underlined in black.

LuZi-4_pET28a(+)

```
atgggcagcagccatcatcatcatcatcacagcagcggcctggtgccgcgcggcagccat
 M   G   S   S   H   H   H   H   H   H   S   S   G   L   V   P   R   G   S   H
atggtgaccggctaccggctctttgaagagatcttaggatccggtgggtccggtggttcc
 M   V   T   G   Y   R   L   F   E   E   I   L   G   S   G   G   S   G   G   S
ggcggcagtggcggttctggaggtagtggatccgggggttccggaggctctggaggttca
 G   G   S   G   G   S   G   G   S   G   S   G   G   S   G   G   S   G   G   S
ggtggttcagggggtagtgtcttcacactcgaagatttcgttggggactgggaacagaca
 G   G   S   G   G   S   V   F   T   L   E   D   F   V   G   D   W   E   Q   T
gccgcctacaacctggaccaagtccttgaacagggaggtgtgtccagtttgctgcagaat
 A   A   Y   N   L   D   Q   V   L   E   Q   G   G   V   S   S   L   L   Q   N
ctcgccgtgtccgtaactccgatccaaaggattgtccggagcggtgaaaatgccctgaag
 L   A   V   S   V   T   P   I   Q   R   I   V   R   S   G   E   N   A   L   K
atcgacatccatgtcatcatcccgtatgaaggtctgagcgccgaccaaatggcccagatc
 I   D   I   H   V   I   I   P   Y   E   G   L   S   A   D   Q   M   A   Q   I
gaagaggtgtttaaggtggtgtaccctgtggatgatcatcactttaaggtgatcctgccc
 E   E   V   F   K   V   V   Y   P   V   D   D   H   H   F   K   V   I   L   P
tatggcacactggtaatcgacggggttacgccgaacatgctgaactatttcggacggccg
 Y   G   T   L   V   I   D   G   V   T   P   N   M   L   N   Y   F   G   R   P
tatgaaggcatcgccgtgttcgacggcaaaaagatcactgtaacagggaccctgtggaac
 Y   E   G   I   A   V   F   D   G   K   K   I   T   V   T   G   T   L   W   N
ggcaacaaaattatcgacgagcgcctgatcacccccgacggctccatgctgttccgagta
 G   N   K   I   I   D   E   R   L   I   T   P   D   G   S   M   L   F   R   V
accatcaacagctccggcgggggtaccatgccgaagcacgagttctctgtggacatgacc
 T   I   N   S   S   G   G   G   T   M   P   K   H   E   F   S   V   D   M   T
tgtggaggctgtgctgaagctgtctctcgggtcctcaataagcttggaggagttaagtat
 C   G   G   C   A   E   A   V   S   R   V   L   N   K   L   G   G   V   K   Y
gacattgacctgcccaacaagaaggtctgcattgaatctgagcacagcatggacactctg
 D   I   D   L   P   N   K   K   V   C   I   E   S   E   H   S   M   D   T   L
cttgcaaccctgaagaaaacaggaaagactgtttcctaccttggccttgagctcattcgt
 L   A   T   L   K   K   T   G   K   T   V   S   Y   L   G   L   E   L   I   R
ggcggatccggcggaagcggcggatccggcggtagcggcggatccggcggctccggcgga
 G   G   S   G   G   S   G   G   S   G   G   S   G   G   S   G   G   S   G   G
tccggcggcagcggcggatccggtggaagcggtggatccggtggtagcggtggatccggt
 S   G   G   S   G   G   S   G   G   S   G   G   S   G   G   S   G   G   S   G
ggaagcggtggatccggtggtagcggtggatccgggggtccgcggatgcagggcacatgc
 G   S   G   G   S   G   G   S   G   G   S   G   G   P   R   M   Q   G   T   C
agtaccactctgattgccattgccggcatgacctgtgcatccagtgtccattccattgaa
 S   T   T   L   I   A   I   A   G   M   T   C   A   S   S   V   H   S   I   E
ggcatgatctcccaactggaaggggtgcagcaaatatcggtgtctttggccgaagggact
 G   M   I   S   Q   L   E   G   V   Q   Q   I   S   V   S   L   A   E   G   T
gcaacagttctttataatcccgctgtaattagcccagaagaactcagagctgctatagaa
 A   T   V   L   Y   N   P   A   V   I   S   P   E   E   L   R   A   A   I   E
gacatgggatttgaggcttcagtcactagtggtggtagttaggggggctccgttaccggc
 D   M   G   F   E   A   S   V   T   S   G   G   S   *   G   G   S   V   T   G
tatcgtctgtttgaaaaagagagcgggggttcgggggggagttggtctcatccacaattt
 Y   R   L   F   E   K   E   S   G   G   S   G   G   S   W   S   H   P   Q   F
gagaagtaa
 E   K   *
```

Figure S8. Nucleotide and amino acid sequence of LuZi-4. Nucleotide and amino acid sequence of LuZi-4 in the pET28a(+) vector. The His-tag and Strep-tag are colored in grey and purple, respectively. The NanoLuc SmallBits, LargeBit, Atox1 and WD4 domains are depicted in light blue, dark blue, red and green, respectively. The restriction sites used for cloning Atox1 and WD4 into the pET28a-CTX-NB-LUMABS plasmid are highlighted in black and bold. The position of the unnatural amino acid pAzF is highlighted in red and bold. The mutation S375C to create LuZi-1 is shown italicized, underlined and in bold. LuZi-6 has the same sequence as LuZi-4, except for the linker between Atox1 and WD4. The linker of LuZi-6 between Atox1 and WD4 is underlined. The optimized LuZi-4.2 and LuZi-6.2 were

created by removing the amino acids highlighted in yellow using overhang PCR and the restriction sites in bold.
